# EXPLORING THE POTENTIAL OF PHLPP INHIBITORS FOR OSTEOARTHRITIS TREATMENT

**DOI:** 10.1093/geroni/igad104.3125

**Published:** 2023-12-21

**Authors:** Leslie Victoria-Sanchez, Haydee Torres, Jennifer Westendorf

**Affiliations:** St. Catherine University, St. Paul, Minnesota, United States; Mayo Clinic, Rochester, Minnesota, United States; Mayo Clinic, Rochester, Minnesota, United States

## Abstract

Osteoarthritis (OA) is a degenerative joint disease characterized by pain, stiffness, and limited joint motion affecting 7% of the world’s population. PH domain leucine-rich repeat protein phosphates (PHLPPs) have been identified as modifiable targets for cartilage regeneration and joint pain. PHLPP1 and PHLPP2 are abnormally expressed in osteoarthritic cartilage and sensory neurons. PHLPP1-/- mice are protected from cartilage degradation and pain-related behaviors (allodynia and reduced mobility) after surgery that destabilizes the medial meniscus (DMM). Here, we investigate the effects of PHLPP inhibitors (i) on human chondrocytes and the effect of Phlpp1 deletion on cell populations within lumbar dorsal root ganglia (DRGs). Immunohistochemistry was performed on mouse DRGs by cryosectioning fixed L3-5 DRGs and using primary antibodies against Fabp7, Anti tubulin beta 3, and PGP9.5/UCHL1. Human chondrocytes were subjected to Cycloheximide chase experiments and treated with PHLPPi (NSC117079 or NSC45586), followed by Western blot analysis to assess protein levels including Phlpp1, Phlpp2, Sox9, p-AKT (Ser473), total AKT, and B-actin, at various time points. We found that PHLPPi prolongs the phosphorylated AKT response when compared to controls and does not affect degradation of Phlpp1 or Phlpp2; additionally, NSC117079 increased production of cartilage extracellular matrix components (glycosaminoglycans and aggrecan) in over 90% of human articular cartilage explants from OA patients. These data advance our understanding of how PHLPPi function within chondrocytes and provide a basis toward understanding their activity and safety in vivo. Our results indicate that PHLPPi NSC117079 is a novel osteoarthritis disease-modifying drug candidate that may have palliative effects.

